# Effect of Silver Doping on the Superconducting and Structural Properties of YBCO Films Grown by PLD on Different Templates

**DOI:** 10.3390/ma15155354

**Published:** 2022-08-03

**Authors:** Ilya A. Shipulin, Aleena Anna Thomas, Sigrid Holleis, Michael Eisterer, Kornelius Nielsch, Ruben Hühne

**Affiliations:** 1 Institute for Metallic Materials, Leibniz-IFW Dresden, 01069 Dresden, Germany; a.anna.thomas@ifw-dresden.de (A.A.T.); k.nielsch@ifw-dresden.de (K.N.); r.huehne@ifw-dresden.de (R.H.); 2 School of Science, TU Dresden, 01062 Dresden, Germany; 3 Atominstitut, TU Wien, Stadionallee 2, 1020 Vienna, Austria; sigrid.holleis@tuwien.ac.at (S.H.); michael.eisterer@tuwien.ac.at (M.E.); 4 Institute of Materials Science, TU Dresden, 01062 Dresden, Germany; 5 Institute of Applied Physics, TU Dresden, 01062 Dresden, Germany

**Keywords:** superconductors, YBCO, thin films, pulsed laser deposition, coated conductor, epitaxy

## Abstract

We report the local structural and superconducting properties of undoped and Ag-doped YBa_2_Cu_3_O_6+x_ (YBCO) films with a thickness of up to 1 µm prepared by pulsed laser deposition on SrTiO_3_ (STO) single crystals and on ion-beam-assisted deposition (IBAD) and rolling-assisted biaxially textured substrate (RABiTS)-based metal templates. X-ray diffraction demonstrates the high crystalline quality of the films on both single crystalline substrates and metal-based templates, respectively. Although there was only a slight decrease in *T_c_* of up to 1.5 K for the Ag-doped YBCO films on all substrates, we found significant changes in their transport characteristics. The effect of the silver doping mainly depended on the concentration of silver, the type of substrate, and the temperature and magnetic field. In general, the greatest improvement in *J_c_* over a wide range of magnetic fields and temperatures was observed for the 5%Ag-doped YBCO films on STO substrates, showing a significant increase compared to undoped films. Furthermore, a slight *J_c_* improvement was observed for the 2%Ag-doped YBCO films on the RABiTS templates at temperatures below 65 K, whereas *J_c_* decreased for the Ag-doped films on IBAD-MgO-based templates compared to undoped YBCO films. Using detailed electron microscopy studies, small changes in the local microstructure of the Ag-doped YBCO films were revealed; however, no clear correlation was found with the transport properties of the films.

## 1. Introduction

Since the discovery of high-temperature superconductors (HTSCs) in the late 1980s [1], great attention has been devoted to the production of affordable flexible conducting HTSC wires with high current density for application in the various fields of technology. For these purposes, mainly second-generation (2G) HTSC tapes based on *RE*Ba_2_Cu_3_O_6+x_ (*RE* = Y or rare earth elements) have been developed in the last decades. Such 2G tapes are manufactured exclusively by thin film technologies, mostly applying the rolling-assisted biaxially textured substrate (RABiTS) [2] or the ion-beam-assisted deposition (IBAD) [3] approach to create a biaxially textured HTSC layer.

Improving the superconducting and transport properties, in particular increasing the critical current density in a wide range of temperatures and magnetic fields, is one of the key issues in the field of applied superconductivity. In order to solve the issue, a large number of approaches and methods have been developed [4,5,6,7,8], among which the controlled creation of defects is still one of the most successful for achieving improved transport characteristics. Since the recent level of technology has reached, at least for the IBAD-based technical templates, an almost single crystalline quality, with an array of small-angle grain boundaries acting as natural defects, such defects are not dense enough to substantially increase the current transport capability for these superconductors in magnetic fields. Therefore, it becomes necessary to introduce artificial defects. Although nanometer-sized oxide precipitates are often used as artificial pinning centers [9,10,11], the addition of silver is an interesting and promising alternative for such defects.

A number of groups have investigated silver as a dopant due to its ambiguous effect on HTSC materials. They showed that silver doping results in quite different behavior, which depends on many factors, such as the growth method [12], concentration of silver [13,14,15], and type of substrate [14,16]. It is currently known that doping silver into YBa_2_Cu_3_O_6+x_ (YBCO) improves the growth properties of the films [13] and enhances the incorporation of oxygen [17,18], which, in turn, can lead to improved transport, mechanical, and even, in some cases, superconducting properties [18,19,20]. In addition, silver has a beneficial influence on the current transport across grain boundaries [14,21,22], which is of considerable interest for metal-based templates due to their granular structure.

Despite the comprehensive study of Ag-doped YBCO, a correlation between the local structure and transport characteristics of Ag-doped YBCO films, in particular on metal-based textured templates such as IBAD-MgO or RABiTS, is still missing. Therefore, the aim of this paper is to summarize our studies on the local structural, superconducting, and transport properties of undoped and Ag-doped YBCO films deposited on SrTiO_3_ single crystals and on IBAD-MgO and RABiTS metal-based templates by pulsed laser deposition. In particular, we used electron backscattering diffraction (EBSD) in a scanning electron microscope to reveal the local grain boundary network of the superconducting layer. At the same time, scanning Hall probe microscopy (SHPM) provided additional information on the local distribution of the critical current density. Similar studies have been performed previously on pure YBCO layers grown on metallic templates [23,24,25].

## 2. Methods

### 2.1. Sample Preparation

For the preparation of the films, we used a pulsed laser deposition (PLD) method with a KrF excimer laser (λ = 248 nm, *Coherent LPXpro 305*, *Göttingen*, *Germany*). Pristine YBCO and Ag-doped YBCO targets with a nominal doping content of 2, 5, and 10 wt.% of silver were synthesized by the conventional solid-state reaction method. The composition of the targets was checked using energy-dispersive X-ray (EDX) spectroscopy on the surface, giving experimental values of 2 ± 0.2 wt.% Ag, 3.8 ± 0.2 wt.% Ag, and 6.4 ± 0.2 wt.% Ag, respectively. Even if the silver concentration of the targets might have been smaller at the surface, we used the nominal concentration in the following. Apart from SrTiO_3_ (STO) single crystals, we used two kinds of technical templates as a substrate: an RABiTS Ni-9 at.%-W tape with a La_2_Zr_2_O_7_/CeO_2_ buffer layers system (provided by *Deutsche Nanoschicht GmbH*, *Rheinbach*, *Germany*) and a Hastelloy tape with an Al_2_O_3_/Y_2_O_3_/IBAD-MgO/MgO/LaMnO_3_/Gd:CeO_2_ buffer layer architecture (provided by *S-Innovations*, *Moscow*, *Russia*). The laser repetition rate was 10 Hz, which corresponds to a growth rate of about 0.8 and 0.6 Å/pulse for undoped and Ag-doped YBCO, respectively. The thickness of the films was estimated using a profilometer and was about 800 nm for all samples presented here. The films were prepared in a 0.4 mbar oxygen atmosphere at a substrate temperature of 810 °C, which was monitored by a thermocouple inside the heater and calibrated by an optical pyrometer. To achieve optimally doped YBCO, subsequent oxygenation under an oxygen partial pressure of 400 mbar at 765 °C completed the process.

### 2.2. Structural Characterization

To check the crystalline structure of the obtained films, X-ray diffraction (XRD) was performed using Co-Kα radiation for standard *θ*–2*θ* scans (*D8 Advance*, *Bruker AXS*, *Karlsruhe*, *Germany*) in Bragg–Brentano geometry and a four-circle goniometer with Cu-K_α_ radiation for the texture investigations *(Panalytical X’pert PW3040*, *Malvern Panalytical*, *Almelo*, *The Netherlands*), respectively. The surface morphology and composition of the films were analyzed by scanning electron microscopy (SEM) in a (*GEMINI 1530*, *LEO/ZEISS*, *Oberkochen*, *Germany*) with an additional energy-dispersive X-ray spectroscopy (EDX) tool *XFlash 6130*, *Bruker Nano GmbH*, *Berlin*, *Germany*).

To study the local structure and the orientation distribution of the grain boundaries, EBSD studies were performed using the same SEM with a (*Nordlys EBSD detector*, *Oxford Instruments NanoAnalysis&Asylum Research*, *High Wycombe*, *United Kingdom*) and the *Oxford Instruments HKL Channel 5* acquisition software. An acceleration voltage of 20 kV, a 2 × 2 binning of the EBSD detector screen, a work distance of 15 mm, and an exposure time of about 0.2 s per EBSD pattern were used to achieve high-quality data. The EBSD mappings were analyzed afterwards with the *MTex* toolbox [26].

### 2.3. Superconducting Characterization

The superconducting and transport properties of the obtained films were measured by a magnetic property measurement system (*MPMS-XL*, *Quantum Design*, *San Diego*, *CA*, *USA*). The superconducting transition temperature (*T_c_*) was determined by measuring the magnetic susceptibility after zero-field cooling (ZFC) and applying a field *µ_0_H* = 0.2 mT along the *c*-axis. The critical current density was determined from hysteretic magnetization curves, which were measured in magnetic fields up to 7 T along the *c*-axis of the crystal structure, using the Bean critical-state model [27]. Remnant field profiles of the films at 77 K were obtained by means of a home-built SHPM setup in a liquid nitrogen bath [28]. The spatial resolution of the obtained field profiles was 50 µm in the x- and y-direction. The local current distributions were calculated from the trapped field profiles using an inversion of Biot–Savart law [29].

## 3. Results and Discussion

In the following section, the structural properties of the grown films are discussed in detail. This includes the surface morphology and the local and global orientation distribution of the YBCO film on the different templates. Afterwards, the superconducting properties are presented with an emphasis on the critical current density *J*_c_. Additionally, global and local measurement techniques are applied in order to check whether a correlation between the structural and superconducting properties can be established. Only selected data are shown in the figure; more data is shown in the Supplementary Materials.

### 3.1. Surface Morphology

**Figure 1:**
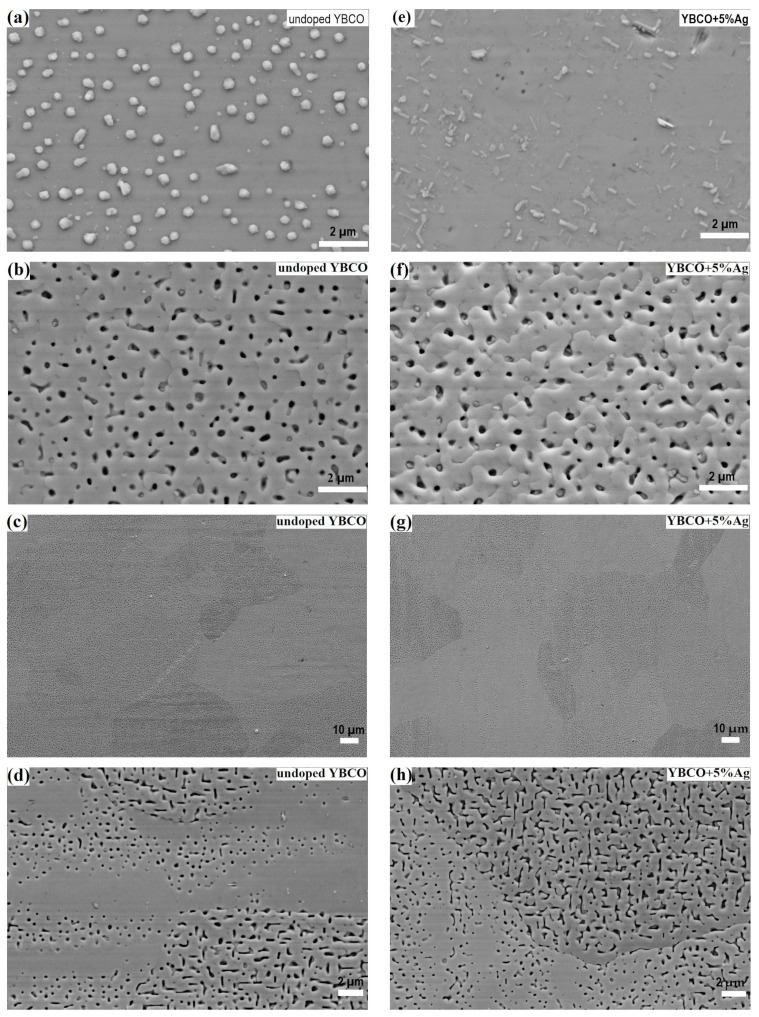
SEM images of the undoped and Ag−doped YBCO films on the (**a**,**e**) STO, (**b**,**f**) IBAD−MgO, and (**c**,**d**,**g**,**h**) RABiTS templates.

The results of the study of the surface morphology are summarized in Figure 1 and Figure S1. The undoped and Ag-doped YBCO films grew homogeneously on the STO substrates, showing only some precipitates (most probably CuO_x_) on the surface (Figure 1a,e). With an increasing silver concentration, the surface became smoother. The 5% Ag-doped YBCO film showed almost no pores but some rectangular-shaped grains, which might be nuclei with an *a*-axis orientation (compare Figure 1e). The films on the IBAD-MgO-based templates also grew rather homogeneously, with small pores and a minor number of particles. The granular structure of the films was visible only at the micrometer scale in the SEM images, as shown in Figure 1b,f. Additionally, an overlap between neighboring grains was observed, leading to a terrace-like surface, which might be explained by the miscut of about 3° from the IBAD-MgO layer. Similar features have been observed for YBCO films grown on miscut substrates [30]. The most pronounced overlap was observed for the 5% Ag-doped YBCO film, as clearly visible in Figure 1f. Finally, the 10% Ag-doped YBCO film had a significant higher defect density (mainly particles, most probably CuO_x_) compared to other Ag-doped films (Figure S1h).

The surface structure of the films on the RABiTS templates was quite different from the other substrates due to the visible granularity arising from large Ni substrate grains with a size of around 50 μm, a high surface porosity, and, in some cases, large misorientation of the grains relative to each other. Figure 1c,d,g,h clearly show a granular surface structure of the films with quite sharp grain boundaries (GBs). Moreover, the porosity of the films varied from grain to grain as a consequence of the individual out-of-plane orientation of the original grain of the metal template [23]. Some grains showed variation in the porosity on one and the same Ni grain, where these features were aligned along the rolling direction (compare Figure 1d). In contrast to the Ag-doped YBCO films on the STO substrates and IBAD-MgO-based templates, no visible changes in the structure and morphology were observed with the increasing Ag content for the films on the RABiTS templates (compare Figure S1).

The composition of the Ag-doped films on the STO substrates, studied with EDX, revealed almost identical values as those identified for the target. However, as is well known, it was difficult to quantify the exact Ag content and the spatial distribution of Ag for these films [13,31].

### 3.2. EBSD Measurements

We used EBSD measurements in order to study the effect of silver doping on the local structure of the films in more detail. This method is a powerful tool for studying the granularity and allows the detection of even minor changes in the local orientation of the grains, analysis of the grain boundaries, etc. Whereas the deposited YBCO films typically replicate the granular structure of the RABiTS- or IBAD-MgO-based templates, the influence of the additional Ag content might result in changes in the local structure, which are detectable by EBSD.

**Figure 2:**
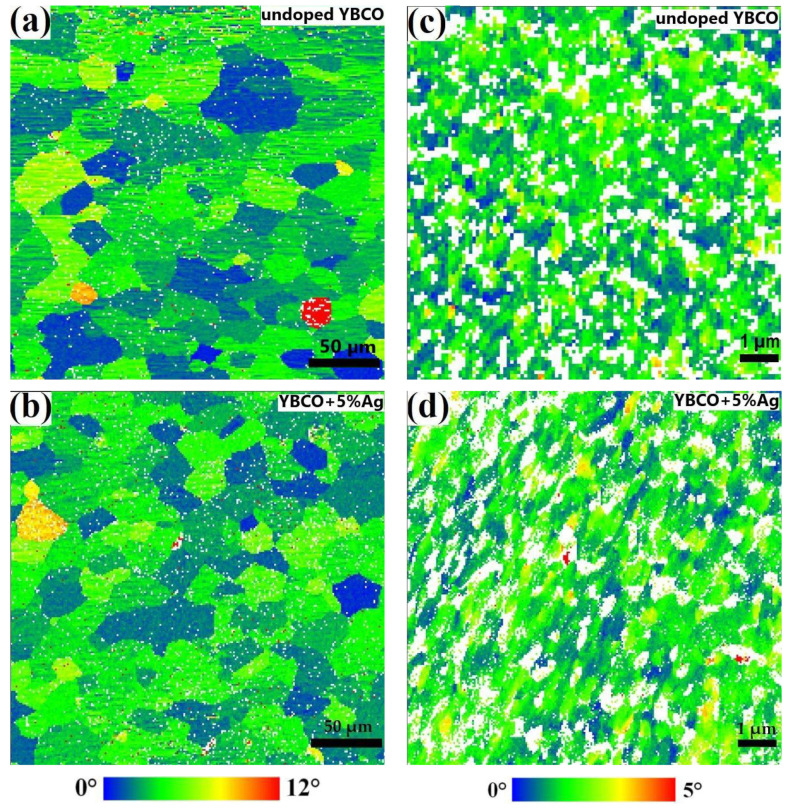
EBSD maps of the absolute misorientation from the ideal cube texture without noise reduction for undoped and Ag-doped YBCO films on (**a**,**b**) RABiTS (step size 1.0 µm) and (**c**,**d**) IBAD−MgO (step size 0.02 µm). White dots are non-indexed areas.

**Figure 3:**
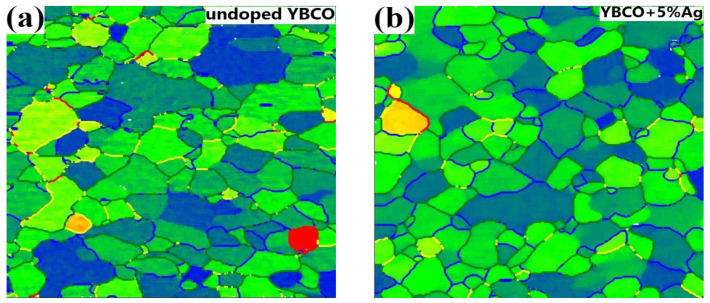

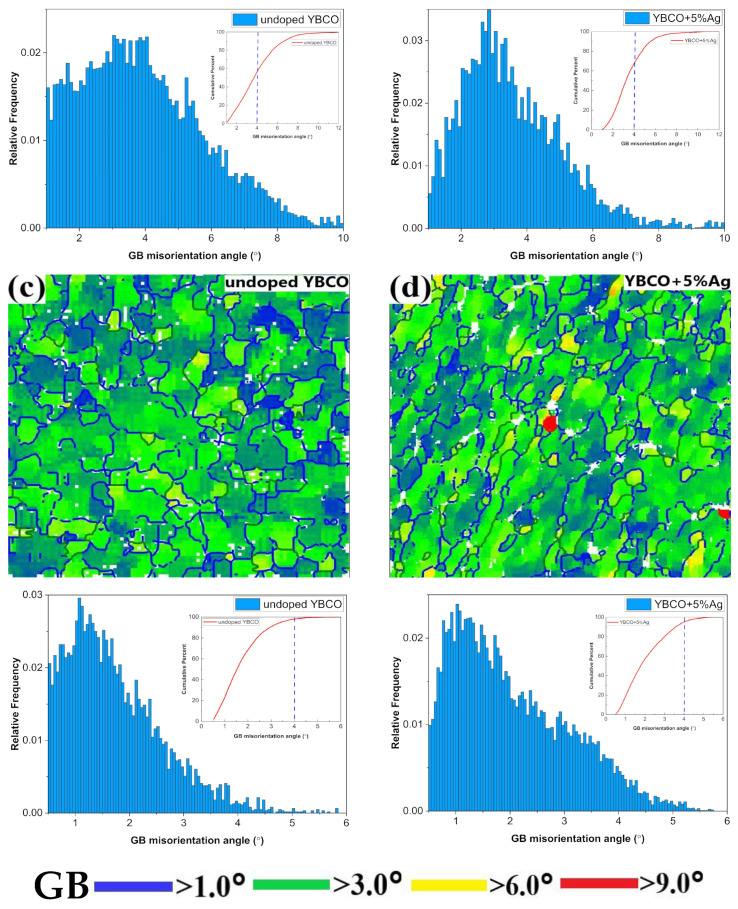
EBSD maps of the absolute misorientation (AMO) with noise reduction and marked grain boundaries for films on RABiTS (**a**,**b**) and IBAD−MgO (**c**,**d**). The grain boundary analysis was carried out with the MTex tool box.

Figure 2a,b and Figure S2a,b show the original EBSD maps for the undoped and Ag-doped YBCO films grown epitaxially on the RABiTS templates. In order to improve the statistical significance of the data, a larger surface area of about 290 µm × 235 µm was additionally investigated, which covers more than 100 original Ni grains. The indexing rate of the Kikuchi patterns was at least 92% for all samples on the RABiTS templates. The majority of the grains were highly textured with an absolute misorientation of less than 8° from the ideal (001)[100] cube orientation. The absolute misorientation angle was calculated from the Euler angles of both grains and refers to the unique rotation (in general, not about one of the crystallographic axes) necessary to align the crystallographic axes of both grains. Only a minor fraction has values greater than 8° (red grains in Figure 2). A statistical analysis was performed on the distribution of the grain boundary misorientation angles as shown in Figure 3 for the undoped and 5%Ag-doped YBCO films. It was necessary to use noise reduction on the original data in order to identify the grain boundaries correctly. The grain boundaries are marked in the images if the misorientation between neighboring measurement points reached a value of 0.5° or 1° for the films on the IBAD-MgO- or RABiTS-based templates, respectively. Finally, the distribution of these misorientations over the misorientation angle was plotted in order to identify the mean value. However, the changes are only on a minor scale as shown in Table 1.

The surface structure of films on IBAD-MgO-based templates differed significantly from the films on the RABiTS templates. Although both templates have a granular structure, the shape, size, and morphology of the grains are fundamentally different. In particular, the films on the IBAD-MgO-based templates are more uniform and, therefore, the granularity of the films was observed only on the micrometer scale [23]. In order to obtain the relevant information on the misorientation distribution, we used a step size of 20 nm for the EBSD map. At the same time, it was not possible to achieve an indexing rate of the Kikuchi pattern above 80%, which is much lower than for the films on the RABiTS templates. This is mainly the result of the porosity on this small scale leading to shadowing effects. As shown in Figure 2c,d and Figure S2c,d, only a few grains had a misorientation angle of 5° and above. In the case of the RABiTS templates, the silver doping had only a minor effect on the GB misorientation angle distribution, i.e., only a slight increase in the mean value was observed (compare Table 1 and Figure 3a,b). Simultaneously, a higher mean value was observed for Ag-doped YBCO layers on IBAD-MgO (Figure 3c,d) but on a significantly lower level compared to the RABiTS-based templates. However, one should keep in mind that the presented data of the GB misorientation angle distribution gives only qualitative rather than quantitative information on the changes associated with Ag doping, since the error in determining the Euler angles might be up to 0.4 degrees, which, in turn, significantly affects the determination of the grain boundary misorientation. The corresponding statistical distributions of the GB misorientation angles are shown under each EBSD map in Figure 3. There are slight variations between the samples on the RABiTS templates; however, one should keep in mind that the data represent only a small part of the sample area. The insets show the cumulative distribution function, which gives the amount of GB below a threshold value. For the samples on the IBAD-MgO-based templates, above 95% of the analyzed GBs showed an MO angle of less than 4°, which is the critical angle for YBCO. For the samples on the RABiTS templates, we found smaller values for the cumulative probability, with about 56% (for undoped and 2%Ag-doped films), 67% (for 5%Ag-doped films), and 45% (for 10%Ag-doped films) considering GBs with an MO angle between 1° and 4°. These grain boundaries should, in principle, not reduce the critical current. The data indicate that silver might have a significantly larger effect on the films on RABiTS than on the IBAD-MgO-based templates, which agrees with the transport data very well.

### 3.3. Structural Characterization Using XRD

**Figure 4:**
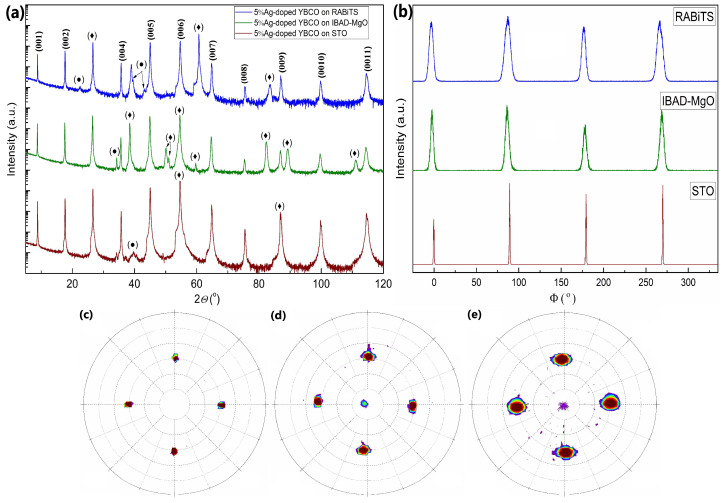
XRD patterns for 5% Ag−doped YBCO films on STO, RABiTS, and IBAD−MgO templates. (**a**) Standard *θ*–2*θ* XRD scans. The symbol (◆) indicates the peaks of the substrate and buffer layers, whereas (●) indicates additional oxides as NiO, NiWO_4_, and Y_2_O_3_; (**b**) in−plane scans of the YBCO (103) reflection. Pole figures of the YBCO (103) planes for the 2%Ag−doped YBCO films on (**c**) STO, (**d**) IBAD−MgO, and (**e**) RABiTS.

The standard XRD patterns shown in Figure 4a indicate a preferential *c*-axis-oriented growth of the Ag-doped films on single crystal substrates and metal-based templates, as only the (00ℓ) peaks of the YBCO phase and the substrate are visible in the *θ*–2*θ* diffraction. Some minor additional peaks were identified as Y_2_O_3_, which is typically incorporated in the YBCO matrix, and NiO or NiWO_4_, resulting from oxidation of the Ni-alloy substrate surface during YBCO processing at higher oxygen partial pressure.

The *c*-axis lattice parameter of the films determined by the Nelson–Riley approximation [32] does not depend on the concentration of silver. The value is about 11.70 Å for the films on the STO substrates and RABiTS templates, and 11.72 Å on the IBAD-MgO based templates, respectively (compare Table 2 for details). In general, the *c*-axis lattice parameters are slightly higher compared to the YBCO films on single crystals (*c* = 11.69 Å) [33] and are in good agreement with previously published data for pure and doped YBCO films [34,35]. Nevertheless, the resulting films have an optimal oxygen concentration since the *c*-axis lattice parameter is similar to that of YBCO with an oxygen content *x* = 1 [36].

In order to determine the preferred out-of-plane orientation (tilt of the *c*-axis), the rocking curves (ω-scans) of the YBCO (005) reflection were measured. The full width at half maximum (FWHM) was ∆ω = 0.3° for the films on the STO substrates. For the films on metal-based templates, the out-of-plane orientation was significantly broader due to the presence of a grain structure and resulted in values of about ∆ω = 2.1° for the films on the IBAD-MgO-based templates and about ∆ω = 4.6° and ∆ω = 8.0° in the longitudinal and transverse direction on the RABiTS templates, respectively. Again, no apparent difference between the undoped and Ag-doped films was found (compare Table 2).

The YBCO (103) plane was used to measure the pole figures and in-plane orientations (φ-scan). The pole figures for the 2%Ag-doped layers in Figure 4c–e show four symmetric peaks, which indicate an epitaxial growth of the films on both the single crystal substrates and metal templates. A slight out-of-plane tilt of a few degrees was observed for the films on the IBAD-MgO-based templates, which is also associated with the production technology of the IBAD layer itself [37]. The film peaks were significantly broader on the RABiTS templates, having an additional anisotropic form due to the different out-of-plane FWHM along the longitudinal (rolling) and transverse direction.

The φ-scans for the films in Figure 4b show sharp reflections every 90° for all substrates, indicating a 4-fold symmetry due to epitaxial growth. As is the case with the out-of-plane orientation, the in-plane FWHM differed significantly for the films on single crystal substrates and metal templates; however, the Ag doping itself seemed to have no influence on the orientation spread. More specifically, the films showed ∆φ = 0.7° on STO, about ∆φ = 4.6° on IBAD-MgO based templates, and about ∆φ = 6.9° on RABiTS templates. Comparing these data with the results of the EBSD measurement, we assume that the relatively large integral FWHM values for the films on the IBAD-MgO-based templates arise from continuous changes in the grain orientation. These gradual changes are visible in some EBSD maps (compare, for example, Figure S2d, where the orientation is continuously changing from top to bottom). In contrast, the sharp grain boundaries for the films on the RABiTS templates indicate an abrupt orientation change from grain to grain. While was is not possible to distinguish between these effects with integral XRD texture measurements, we clearly observed these differences in the EBSD measurements.

### 3.4. Superconducting Properties

**Figure 5:**
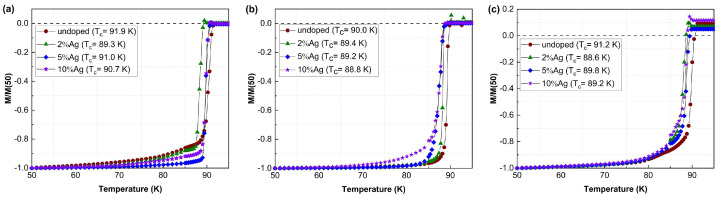
Temperature dependence of the normalized magnetization for undoped and Ag−doped YBCO films on (**a**) STO, (**b**) IBAD−MgO, and (**c**) RABiTS.

The superconducting transition temperature (*T_c_*) and the critical current density (*J_c_*) of the films were determined by magnetization measurements. Figure 5 shows the temperature dependence of the normalized magnetization for undoped and Ag-doped YBCO films on the STO substrates and the RABiTS- and IBAD-MgO-based templates. The superconducting transition temperature was measured after zero field cooling (ZFC) at *µ_0_H* = 0.2 mT along the *c*-axis.

*T_c_* was determined from the onset of the diamagnetic signal. The highest *T_c_* of about 91 K was observed for the undoped and Ag-doped YBCO films grown on STO substrates as shown in Figure 5a. Among the doped films, the 5%Ag-doped sample showed the highest *T_c_* with a sharp transition. Figure 5b shows the *T_c_* data for the films on the IBAD-MgO-based templates. The dependence on doping is almost identical to the films on STO substrates, i.e., the undoped YBCO showed the highest *T_c_* with about 90 K, whereas the 5%Ag-doped films had the highest transition temperature for the doped samples. However, the *T_c_* was at least 2 K smaller compared to the corresponding films on STO substrates.

The results for the undoped and Ag-doped YBCO films on the RABiTS templates are shown in Figure 5c. A high *T_c_* was observed for undoped YBCO with about 91.2 K, which is only slightly lower than the value on the STO substrates. However, the transition width was significantly larger compared to the films on the other substrates, which might be associated mainly with the presence of granularity. In general, the films on the RABiTS templates had slightly higher *T_c_* compared to the films on the IBAD-MgO-based templates, although the texture of the films on IBAD-MgO was significantly sharper. At the same time, the films on the RABiTS templates had almost the same *c*-axis lattice parameter as the films on STO, which might be the reason for the high *T_c_*. Furthermore, there was a slight decrease in *T_c_* of about 1–2 K with silver doping. The detailed results are summarized in Table 3. 

**Figure 6:**
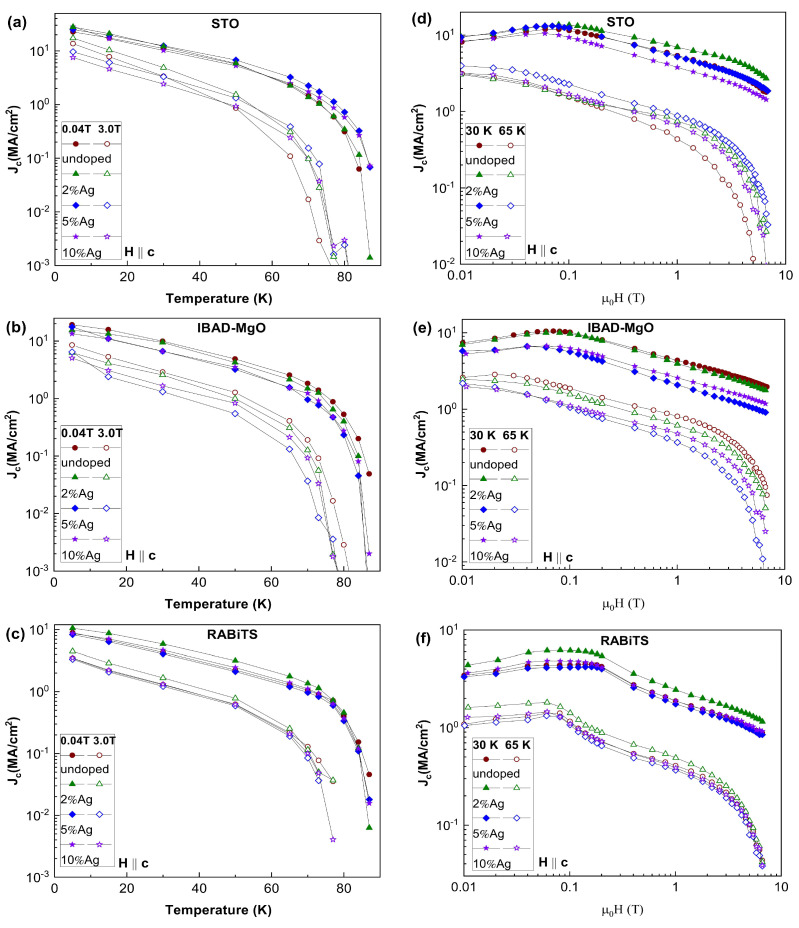
Dependence of the critical current density *J_c_* on the (**a**–**c**) temperature and (**d**–**f**) magnetic field for undoped and Ag−doped YBCO films deposited on different templates.

The critical current density (*J_c_*) of the films was calculated from the magnetization hysteresis loops using the Bean model. The temperature and magnetic field dependences of *J_c_* are shown in Figure 6 for selected parameters; additional results are summarized in Figures S3 and S4. The highest *J_c_*_,0_ values for the Ag-doped YBCO were observed in the films on the STO substrates, where a significant improvement was found compared to the undoped samples. A similar trend was observed for the doped films on the RABiTS-based templates. In contrast, no improvement was found for the doped YBCO films on the IBAD-MgO-based templates for all temperatures and magnetic fields studied. The *J_c_*_,0_ values at 77 K of the undoped and Ag-doped YBCO films on the STO substrates and metal-based templates are included in Table 3.

The Ag-doped YBCO films on the STO substrates showed the highest *J_c_* values over the entire range of temperatures and magnetic fields. In general, the silver doping improved the *J_c_* values on the STO substrates compared to the undoped films, which is in good agreement with some published data [20,31]. In fact, 5%Ag seemed to be an optimal concentration for the YBCO films on STO substrates in a wide range of temperatures and magnetic fields, as shown in Figure 6a and Figure S3a. In particular, the 2%Ag-doped YBCO films on STO substrates showed the highest *J_c_* value at 77 K. However, for temperatures of 30 K and below, the critical current density of the 2%Ag-doped YBCO films on STO was higher for fields above *µ*_0_*H* = 0.1 T, as clearly shown in Figure 6d and Figure S3d. 

In the case of the films on the IBAD-MgO-based templates, the silver doping did not result in any improvement of the transport characteristics. On the contrary, *J_c_* decreased for the Ag-doped YBCO films on the IBAD-MgO-based templates over the entire range of temperatures and magnetic fields, as shown in Figure 6b,e and Figure S3b,e. The maximum *J_c_*_,0_ at 77 K on this type of template reached 1.8 MA/cm^2^ for the undoped YBCO film, which is slightly higher than for the undoped YBCO film on the STO substrate. 

A slightly different situation was observed for the Ag-doped YBCO films on the RABiTS templates. Actually, the silver doping improved the *J_c_* values but only for 2%Ag and mainly at temperatures below 65 K. Above this temperature, the dependence is almost similar to the data for the films on the IBAD-MgO-based templates, i.e., the undoped YBCO films showed superior transport properties, especially at *µ*_0_*H* > 1.0 T, as shown in Figure 6c and Figure S3c. The *J_c_*_,0_ values at 77 K on this type of substrate were slightly lower compared to the other substrates and, at best, was about 0.9 MA/cm^2^ for the 2% Ag-doped films.

### 3.5. SHPM Measurements

For a more detailed study of the critical current density, we used scanning Hall probe microscopy. The main advantage of this method is the spatial-resolved imaging of the magnetic properties in the samples. This yields information on the local differences of the superconducting properties in the grown films such as defects and inhomogeneities.

**Figure 7:**
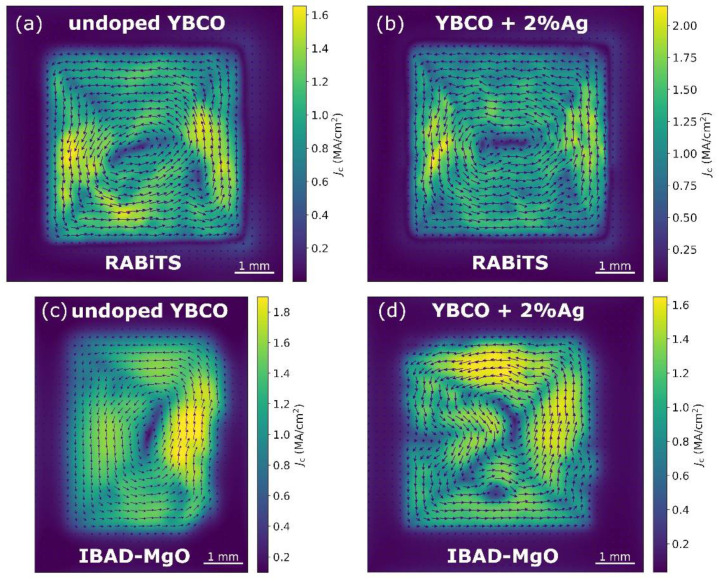
Local critical current density distribution of the undoped and Ag−doped YBCO films calculated from SHPM maps on (**a**,**b**) RABiTS and (**c**,**d**) IBAD−MgO. The sample size was 5 mm × 5 mm.

Figure 7 shows the distribution of the local critical current density at 77 K in the self-field with a spatial resolution of 50 µm for undoped and Ag-doped YBCO films on different templates. For the 2%Ag-doped film on the RABiTS template, as shown in Figure 7b, we observed a slight increase in the local critical current density to 2 MA/cm^2^ in comparison with the respective undoped film. On the contrary, a decrease in the local *J_c_* was found for the Ag-doped films on the IBAD-MgO-based templates, as shown in Figure 7c,d and Figure S4c,d. It should be noted that the data on the critical current density distribution correlate well with the results of the global magnetization measurements presented above. In particular, the increase in the local critical current density for the 2%Ag-doped film on the RABiTS template (Figure 7b) was also observed for the SQUID data in low magnetic fields, as shown in Figure S3c. Furthermore, for the Ag-doped films on the IBAD-MgO-based templates, the data obtained from the SHPM measurements fully correlate with the transport data shown in Figure S3b, i.e., the *J_c_* of the undoped YBCO films was superior to the Ag-doped YBCO films in low magnetic fields. With this spatial resolution, the magnetic granularity appeared to be similar for all films on RABiTS, independently of their doping. In order to investigate the effect of granularity on the transport properties, further SHPM measurements at different temperatures at a higher resolution are needed.

### 3.6. Discussion of the Results

In general, the obtained results indicate that the influence of silver might be fundamentally different for the different types of substrates. The most ambiguous effect of silver was observed for the films on the RABiTS templates, where the silver improved the transport characteristics in a certain range of temperatures and magnetic fields. Currently, we do not have a complete understanding of this effect, but we suppose it might be associated with the creep in the magnetic flux below 65 K, where the probability of thermal activation of vortices decreases and the pinning force becomes much stronger and mainly depends on the type of pinning centers. According to the transport data, we can only conclude that the silver doping might alter the effect of existing pinning centers or acts as additional pinning centers. However, it remains unclear exactly what role silver plays in this process.

Assuming that the silver does not induce pinning centers, it might penetrate into the grain boundaries, changing the transport properties by forming superconductor-normal metal-superconductor (S-N-S) junctions instead of existing superconductor-isolator-superconductor (S-I-S) junctions. In this case, the dopant does not directly participate in the pinning but allows vortices to move between grains more freely, thus we observed a sharper drop in the *J_c_* with the increasing magnetic field (when the vortex density increases). However, as the temperature decreases, the probability of thermal activation of vortices also decreases and the contribution from this effect gradually decreases. Similar assumptions were made by Aylin Yildiz et al. [21] and Pinto R. et al. [31] for bulk materials and for films on single crystal substrates.

The assumption of a S-I-S to S-N-S transition may be particularly valid for films on metal-based templates showing larger GB misorientation angles. To check for this, we used the temperature dependence of the critical current density, as shown in Figure 6a–c, which was fitted with an expression $J_{c}\propto {\left(1-t\right)}^{q}$, where t=TTc. The exponent *q* should be equal to 3/2 and 2 in the framework of the Ginzburg–Landau theory for S-I-S junctions [38] and the de Gennes–Werthamer–Clarke theory for S-N-S junctions [39,40,41], respectively. It was found that for undoped and Ag-doped YBCO films on STO, the power *q* was about 2, which indicates the presence of S-N-S junctions, whereas for films on metal-based templates, the power *q* changed slightly but mainly stayed at about 3/2, which in turn indicates the presence of S-I-S junctions, as shown in Table 3. Thus, silver doping of YBCO films, both on STO substrates and metal-based templates, did not lead to the suggested change in the transitions between S-I-S and S-N-S.

Nevertheless, an improvement in the transport characteristics of Ag-doped YBCO films on STO substrates and, to a lesser extent, on RABiTS templates was obvious. Our detailed X-ray diffraction studies and studies of the surface morphology and local microstructure showed some changes in the local microstructure for Ag-doped films, in particular the GBs misorientation angle. However, these changes turned out to be quite small, and, therefore, we assume that there is no explicit correlation between the local GB distribution and the observed changes in the transport characteristics. Moreover, it remains unclear why the optimal concentration of silver for the films on the STO substrates and RABiTS templates depends on the range of temperatures and magnetic fields. Nevertheless, with regard to the Ag-doped YBCO films on STO substrates, it is most likely that the major role of silver is related to the improvement in the film growth process, since we only found changes in the surface morphology of the films in the form of significantly decreased CuO_x_ precipitates. Additional studies using a higher-resolution SHPM method or detailed transmission electron microscopy investigation of GB may shed light on some of the issues mentioned above and make it possible to understand the real mechanism of silver doping.

## 4. Conclusions

For the first time, we performed a detailed study on the local structural, superconducting, and transport properties of Ag-doped YBCO films grown on STO substrates and on RABiTS- and IBAD-MgO-based templates using pulsed laser deposition. The X-ray diffraction studies demonstrated the high crystalline quality of the films on both single crystal substrates and metal-based templates. Some changes in the surface morphology were observed only for the Ag-doped YBCO films on the STO substrate while no obvious differences were found on the metal-based templates. Based on the EBSD measurements, small changes in the local structure, in particular the GBs misorientation angle distribution, were detected for both types of metal-based templates. Measurements of the superconducting properties showed a slight decrease of about 1–2 K in the superconducting transition temperature for the Ag-doped YBCO films on all templates. Studies of the transport properties indicated that the effect of silver doping is ambiguous. The highest critical current density was found for a silver concentration of 2% for the films on the RABiTS templates, whereas 2% or 5% Ag resulted in the best properties on the STO substrates depending on the range of temperatures and magnetic fields. In contrast, no improvement was observed for doped YBCO films on the IBAD-MgO-based templates. A local study of the current distribution was performed using SHPM measurements, showing good agreement with the global transport characteristics. However, the resolution of these local studies was not sufficient to draw a final conclusion on the correlation between the changes in the local microstructure (i.e., slight variations in the GBs’ misorientation angle distribution) and the transport characteristics.

## Figures and Tables

**Table 1:** Mean values for the grain boundaries’ misorientation distribution determined from EBSD measurements for undoped and Ag−doped YBCO films grown on RABiTS and IBAD−MgO-based templates.

	GBs Misorientation, Degree	
	IBAD-MgO	RABiTS
undoped	1.6 (98.2%) *	3.8 (56.7%)
2% Ag	1.5 (98.8%)	3.8 (56.6%)
5% Ag	1.8 (95.2%)	3.3 (67.3%)
10% Ag	1.7 (97.0%)	4.3 (45.6%)

* Value of cumulative probability for GB misorientations up to 4°.

**Table 2:** Structural parameters determined from XRD measurements for undoped and Ag−doped YBCO films grown on different templates. For the RABiTS templates, the first value for ∆ω is along the rolling direction and the second perpendicular to it.

Substrate	YBCO Lattice *c* (Å)	∆*φ* (103) (°)	∆*ω* (005) (°)
Undoped YBCO			
STO	11.701	0.7	0.3
RABiTS	11.705	6.8	4.6/8.0
IBAD-MgO	11.714	4.7	2.1
YBCO + 2%Ag			
STO	11.699	1.1	0.3
RABiTS	11.703	6.8	4.8/7.4
IBAD-MgO	11.707	4.6	2.0
YBCO + 5%Ag			
STO	11.696	0.8	0.3
RABiTS	11.704	6.5	4.8/7.1
IBAD-MgO	11.722	3.8	2.1
YBCO + 10%Ag			
STO	11.700	1.0	0.3
RABiTS	11.703	7.1	4.6/8.0
IBAD-MgO	11.718	4.6	2.1

**Table 3:** Superconducting properties of undoped and Ag−doped YBCO films grown on different templates. *T_c_* was determined from magnetization measurements, whereas *J_c_*_,0_ was determined at *H* = 0, and *q* was estimated from the fitting of the temperature dependence of *J_c_*.

Substrate	*T_c_* (K)	*J*_*c*,0_ at 77 K (MA/cm^2^)	*q*
Undoped YBCO			
STO	91.9	1.4	2.4
RABiTS	91.2	0.9	1.7
IBAD-MgO	90.0	1.8	1.7
YBCO + 2%Ag			
STO	89.3	2.5	2.2
RABiTS	89.4	1.1	1.5
IBAD-MgO	88.6	1.5	1.6
YBCO + 5%Ag			
STO	91.0	2.0	1.9
RABiTS	89.8	0.8	1.7
IBAD-MgO	89.2	1.4	1.5
YBCO + 10%Ag			
STO	90.7	2.0	2.6
RABiTS	89.2	0.8	1.5
IBAD-MgO	88.8	1.4	1.8

## Data Availability

All data in this work are available on request by contact with the corresponding author.

## Supplementary Materials

The following supporting information can be downloaded at: https://www.mdpi.com/article/10.3390/ma15155354/s1, Figure S1: SEM images of the undoped and Ag-doped YBCO films; Figure S2: EBSD maps of the absolute misorientation for undoped and Ag-doped YBCO films; Figure S3: Dependence of the critical current density *J_c_* in the temperature and magnetic field; Figure S4: Local critical current density distribution calculated from SHPM maps.
