# Supplementary material for: Effect of Silver Doping on the Superconducting and Structural Properties of YBCO Films Grown by PLD on Different Templates
Source: Materials (Basel). 2022 Aug 3;15(15):5354. doi: 10.3390/ma15155354 (PMC9369561; doi:10.3390/ma15155354)
Supplement: Supplementary file 1 [file materials-15-05354-s001.zip › materials-1816783-supplementary.pdf]

materials

MDPI

Article

Effect of Silver Doping on the Superconducting and Structural Properties of YBCO Films Grown by PLD on Different Templates

Ilya A. Shipulin [1,2,*], Aleena Anna Thomas [1,2], Sigrid Holleis [3], Michael Eisterer [3], Kornelius Nielsch [1,4,5] and Ruben Hühne [1]

1 Institute for Metallic Materials, Leibniz-IFW Dresden, 01069 Dresden, Germany
2 School of Science, TU Dresden, 01062 Dresden, Germany
3 Atominstitut, TU Wien, Stadionallee 2, 1020 Vienna, Austria
4 Institute of Materials Science, TU Dresden, 01062 Dresden, Germany
5 Institute of Applied Physics, TU Dresden, 01062 Dresden, Germany
* Correspondence: i.shipulin@ifw-dresden.de or shipuliniliya@gmail.com

Surface morphology

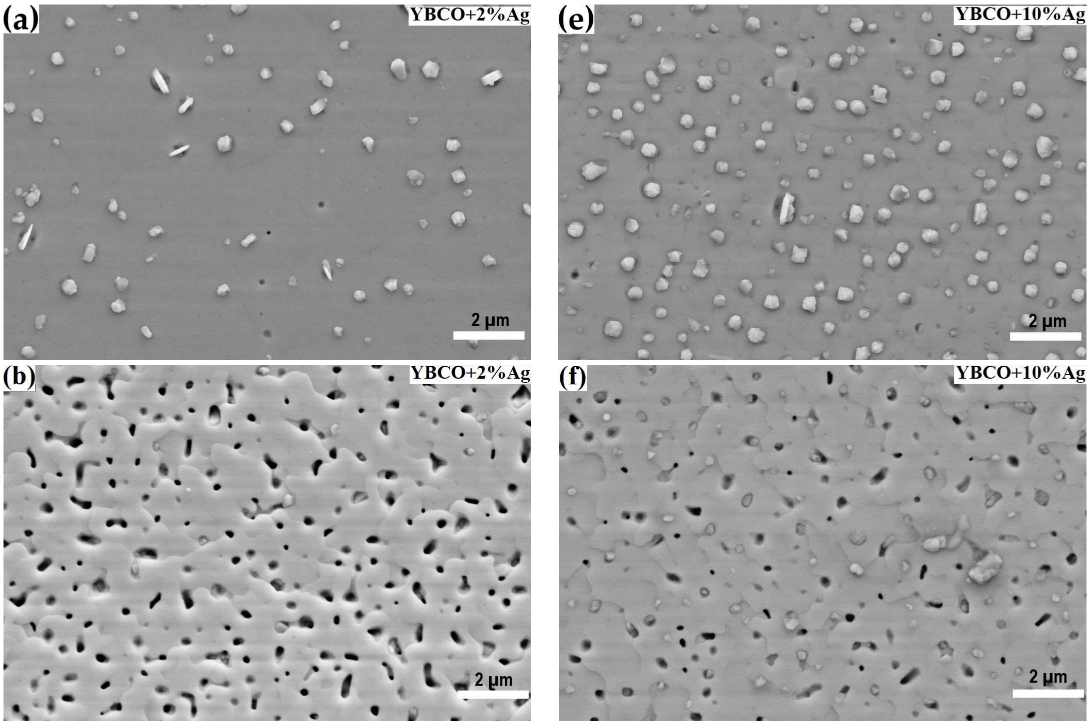

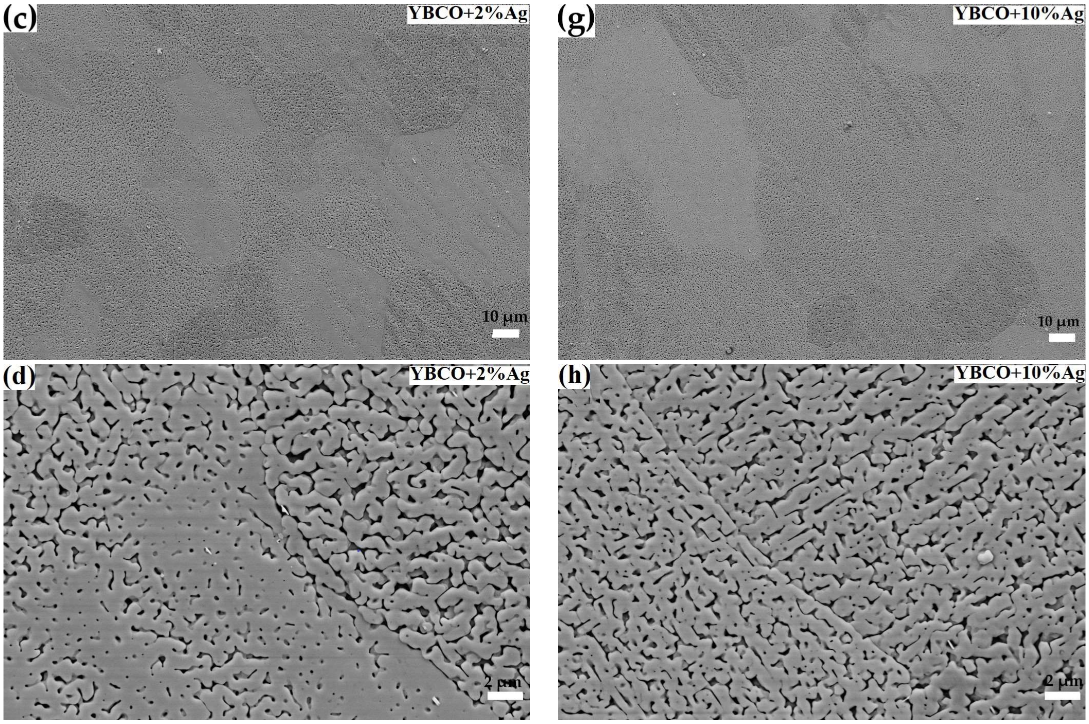

Figure S1. SEM images of the undoped and Ag–doped YBCO films on **(a)**, **(e)** – STO, **(b)**, **(f)** – IBAD–MgO, and **(c,g)**, **(d,h)** – RABiTS templates.

EBSD measurements

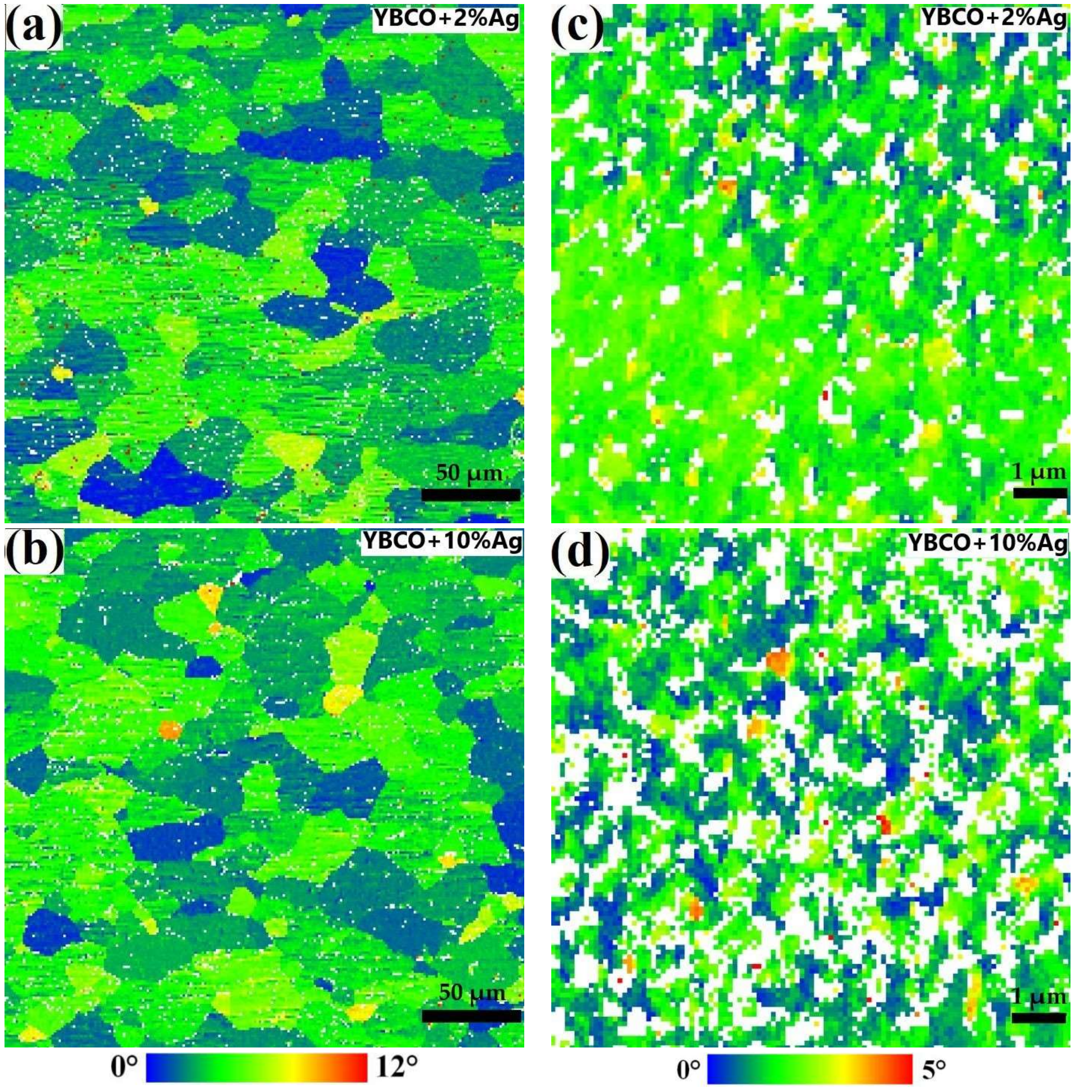

Figure S2. EBSD maps of the absolute misorientation from the ideal cube texture without noise reduction for undoped and Ag−doped YBCO films on **(a-b)** – RABiTS (step size 1.0 μm) and **(c-d)** – IBAD−MgO (step size 0.02 μm). White dots are non-indexed areas.

Transport properties

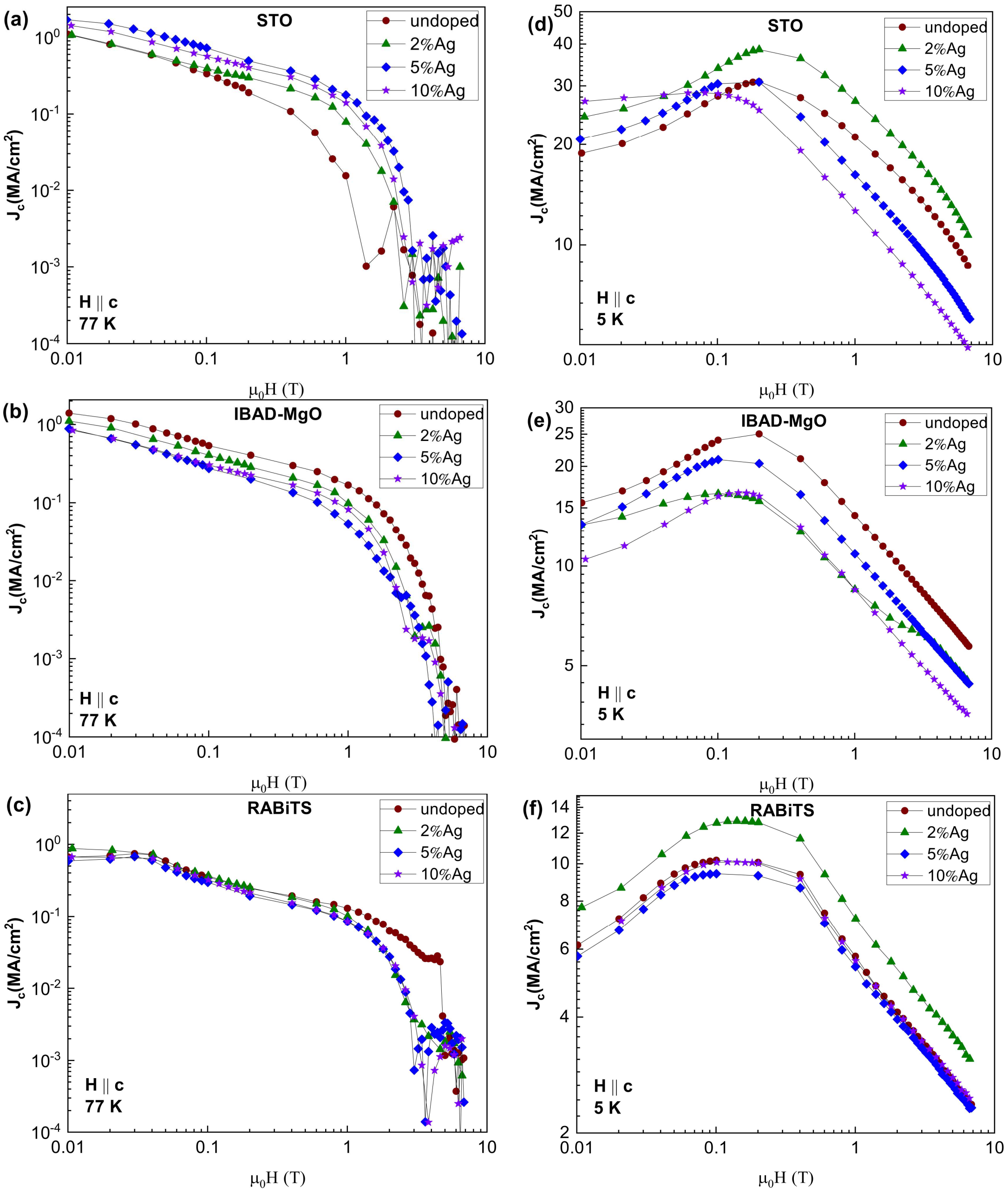

Figure S3. Dependence of the critical current density J_c on: **(a–c)** temperature and **(d–f)** magnetic field for undoped and Ag–doped YBCO films deposited on the different templates.

SHPM measurements

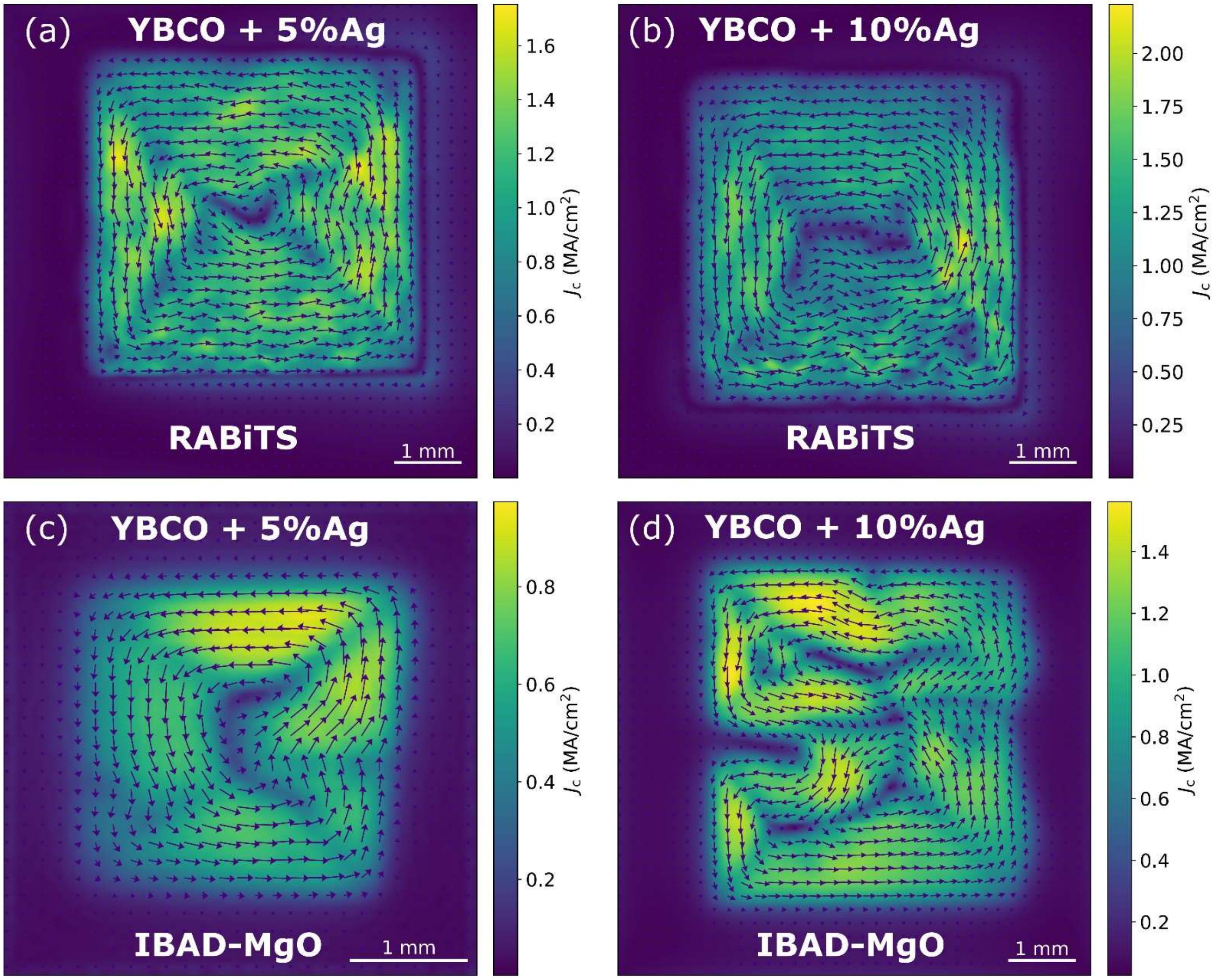

Figure S4. Local critical current density distribution of the undoped and Ag–doped YBCO films calculated from SHPM maps on: **(a-b)** RABiTS and **(c-d)** IBAD–MgO.
